# Linking Cell Polarity to Cortical Development and Malformations

**DOI:** 10.3389/fncel.2019.00244

**Published:** 2019-06-04

**Authors:** Janne Hakanen, Nuria Ruiz-Reig, Fadel Tissir

**Affiliations:** Université catholique de Louvain, Institute of Neuroscience, Developmental Neurobiology, Brussels, Belgium

**Keywords:** neurogenesis, neuronal migration anomalies, axon and dendrite polarization, synapse formation, axon growth and guidance

## Abstract

Cell polarity refers to the asymmetric distribution of signaling molecules, cellular organelles, and cytoskeleton in a cell. Neural progenitors and neurons are highly polarized cells in which the cell membrane and cytoplasmic components are compartmentalized into distinct functional domains in response to internal and external cues that coordinate polarity and behavior during development and disease. In neural progenitor cells, polarity has a prominent impact on cell shape and coordinate several processes such as adhesion, division, and fate determination. Polarity also accompanies a neuron from the beginning until the end of its life. It is essential for development and later functionality of neuronal circuitries. During development, polarity governs transitions between multipolar and bipolar during migration of postmitotic neurons, and directs the specification and directional growth of axons. Once reaching final positions in cortical layers, neurons form dendrites which become compartmentalized to ensure proper establishment of neuronal connections and signaling. Changes in neuronal polarity induce signaling cascades that regulate cytoskeletal changes, as well as mRNA, protein, and vesicle trafficking, required for synapses to form and function. Hence, defects in establishing and maintaining cell polarity are associated with several neural disorders such as microcephaly, lissencephaly, schizophrenia, autism, and epilepsy. In this review we summarize the role of polarity genes in cortical development and emphasize the relationship between polarity dysfunctions and cortical malformations.

## Introduction

Polarity describes structural, biochemical, or functional asymmetry in cells. All mammalian cells experience polarity during their lifespan. In some cases, the polarization process is transient, occurs in response to mechanical or biochemical stimuli, and results in reorganization of the plasma membrane and cytoskeleton. In other cases such as in epithelial and neural cells, the polarity is permanently needed to achieve cellular fates and functions. There are two type of polarity: the cell intrinsic apical–basal polarity and the tissue wide polarity known as planar cell polarity.

## Apical–Basal Polarity

**FIGURE 1:**
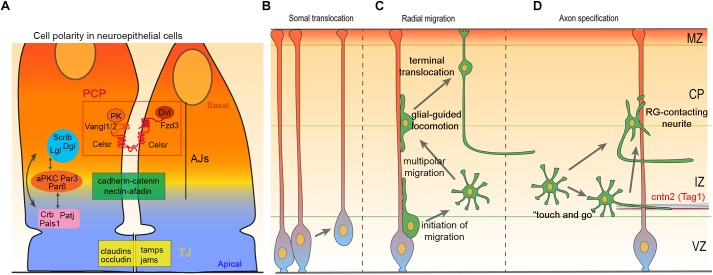
Cell polarity, migration and axon specification in cortical development. **(A)** Schematic illustration of neuroepithelial apical junctional complexes: tight junction (TJ) and adherens junction (AJ) define apical–basal polarity and delineate cellular domains. These junctions regulate cell–cell adhesion, cellular transport and serve as an intracellular anchoring site for apical–basal polarity complexes, Crb-Pals1-PatJ, Par3-Par6-aPKC, and Scrib-Lgl-Dlg. Apical–basal polarity complexes interact with each other and with PCP proteins Celsr1-3, Fzd3/6, Vangl1/2, Dvl1-3, and Pk1-4, to establish and maintain cell polarity. **(B–D)** Diagram illustrating somal translocation **(B)**, multi-phase radial migration **(C)**, and axon specification in the cerebral cortex **(D)**.

Apical–basal polarity divides the cytoplasm and plasma membrane into distinct functional domains. This is particularly important for epithelial cells lining the lumen of internal cavities in organs and tissues. In the central nervous system, neuroepithelial cells become polarized during neurulation when neural plate undergoes a series of morphological changes to form the neural tube. The apical domain of neuroepithelial cells localizes adjacent to the fluid-filled ventricular lumen and basal domain extends radially to the outer surface of the neural tube (Colas and Schoenwolf, 2001). This organization, with specialized apical and basal domains is later found in apical radial glia (aRG) in cerebral cortex. To achieve apical–basal polarity, neuroepithelial cells and aRG require protein trafficking and formation of polarized junctional complexes. Apico-lateral boundary of the neuroepithelial cells and aRG have two type of junctions that form a belt-like structure called apical junctional complexes (AJCs): Tight junctions (TJs) form a boundary that ensures leak-tightness and prevent the passage of molecules and ions in the space between cells (Figure 1A). TJs are composed of claudins, tight junctions-associated MARVEL proteins (TAMPs, occludin/tricellulin/marvelD3) and adhesion molecules. Adherens junctions (AJs) mediate intercellular binding of adjacent cells on the extracellular side and interact with the cell cytoskeleton on the intracellular side. AJs major components are cadherin–catenin and nectin–afadin complexes (Figure 1A) (Cording et al., 2013; Mori et al., 2014; Rodriguez-Boulan and Macara, 2014; Campanale et al., 2017; Chou et al., 2018). Thus, AJCs serve as major interacting points between epithelial cells and determine their apical–basal domains. The intracellular trafficking machinery regulates vesicle formation, sorting and transport of membrane-bound proteins. Apical–basal oriented trafficking ensures the polarized distribution of proteins into apical and basal domains. Formation and maintenance of the AJCs and polarized trafficking is a subtle interactive process guided by polarity proteins (Cao et al., 2012; Rodriguez-Boulan and Macara, 2014; Roman-Fernandez and Bryant, 2016). Major polarity protein complexes include the Partitioning defective proteins (Par)3, Par6, and atypical protein kinase C (aPKC), Crumbs (Crb) proteins associated with Lin-7 1 (Pals1) and Pals1 associated tight junction protein (PatJ), and Scribble (Scrib)-Lethal giant larvae (Lgl)-Discs large, (Dlg) complexes (Figure 1A) (Rodriguez-Boulan and Macara, 2014; Roman-Fernandez and Bryant, 2016; Campanale et al., 2017; Chou et al., 2018). The functional knowledge of Par3-Par6-aPKC, Crb-Pals1-Patj1, and Scribble -Lgl-Dlg polarity complexes in apical–basal polarity originates predominantly from studies in *Caenorhabditis elegans* and *Drosophila melanogaster* (Kemphues et al., 1988; Wodarz et al., 1995; Bilder and Perrimon, 2000). Par3-Par6-aPKC, Crumbs-Pals1-Patj1, and Scribble-Lgl-Dlg polarity complexes functionally interact with each other and vast number of proteins related to the cell polarization including components of the planar cell polarity (Figure 1A).

## Planar Cell Polarity

Planar cell polarity (PCP), also referred to as tissue polarity, designates the organization of cells along the planar axis orthogonal to the apicobasal axis. PCP is critical for the fidelity of embryonic development and the proper shaping of multicellular tissues in different organisms (Axelrod and McNeill, 2002; Jenny and Mlodzik, 2006; Jones and Chen, 2007; Wang and Nathans, 2007; Adler, 2012; Tissir and Goffinet, 2012; Butler and Wallingford, 2017). The establishment of PCP relies on two molecularly independent pathways. The Fat (Ft; Fat1–4 in mammals), Dachsous (Ds; Dchs1 and 2 in mammals), and Four-jointed (Fj; Fjx1 in mammals) pathway (Blair and McNeill, 2018), and the “core” PCP pathway. The core PCP module includes the following transmembrane proteins: The Flamingo/Starry night cadherin (Fmi/Stan; Celsr1–3 in mammals), Frizzled (Fz; Fzd3 and 6 in mammals), and Van Gogh/Strabismus (Vang/Stbm; Vangl1 and 2 in mammals) that communicate polarity between cells, and cytoplasmic proteins Disheveled (Dsh; Dvl 1–3 in mammals), and Prickle (Pk; Pk1–4 in mammals) that amplify the intracellular asymmetries and convert them into changes in cytoskeleton, cell shape, and behavior. In response to mechanical forces and WNT ligands gradients, core PCP proteins transiently assemble into two mutually exclusive complexes at opposite sides of cells (i.e., Celsr/Fzd/Dvl at one side, and Celsr/Vangl/Pk at the facing side), creating a uniform pattern throughout the whole tissue. Symmetry breaking is triggered by microtubule-mediated trafficking and different signaling pathways that cooperatively regulate the localization and stabilization of PCP components at discrete regions of the cell membrane. In mammals, PCP is involved in a constantly growing number of developmental processes and is required for the proper function of several organs. Typical examples include (not limited to) the orientation of inner ear hair cells (Wang Y. et al., 2006; Kelly and Chen, 2007; Ezan and Montcouquiol, 2013; Duncan et al., 2017), of cell division in kidney tubules (Bacallao and McNeill, 2009; McNeill, 2009; Carroll and Yu, 2012; Goggolidou, 2014; Papakrivopoulou et al., 2014), and of skin hair follicles (Guo et al., 2004; Devenport and Fuchs, 2008; Ravni et al., 2009); patterning of the papillae on the tongue (Wang Y. et al., 2016); positioning and directional beating of cilia in the embryonic node (Antic et al., 2010), oviduct (Shi et al., 2014; Shi et al., 2016), and trachea (Vladar et al., 2012; Tsuji et al., 2018); morphogenesis of cardiac and lymphatic valves (Tatin et al., 2013; Gibbs et al., 2016); as well as the differentiation of beta cells and glucose homeostasis (Cortijo et al., 2012). In the nervous system, PCP is crucial for neural tube closure (Ueno and Greene, 2003; Tissir and Goffinet, 2010; Allache et al., 2012; Robinson et al., 2012; Tissir and Goffinet, 2013b), tangential migration of facial branchiomotor neuron in the hindbrain (Wada and Okamoto, 2009; Qu et al., 2010; Sittaramane et al., 2013; Glasco et al., 2016), and polarity of ependymal cilia (Tissir et al., 2010; Boutin et al., 2012, 2014; Ezan and Montcouquiol, 2014).

Even though apical–basal polarity and planar cell polarity have been studied separately, increasing evidence suggests that they share components and downstream effectors, and interact with each other in networks of positive and negative feedback loops that coordinate cell behavior. In this review we focus on core apical–basal and planar cell polarity complexes in development and function of the mammalian cerebral cortex.

## Apical–Basal Polarity in Cortical Neurogenesis

At the initial stages of forebrain development, the dorsal telencephalon is organized in a pseudostratified epithelium consisting of neural stem cells (also known neuroepithelial cells) that undergo multiple rounds of symmetric division to expand the initial pool of progenitors. Once neurogenesis begins, the neocortex comprises two germinal zones: The ventricular zone (VZ) lining the lateral ventricles and containing aRG, also referred to as apical neural progenitor cells (aNPC), and the adjacent subventricular zone (SVZ), located dorsally to the VZ and containing intermediate/basal progenitors (IP/BP). In VZ, aRG undergo several rounds of divisions to self-renew and generate excitatory neurons. aRG also give rise to IP/BP cells and basal radial glia (bRG, especially in primates), which delaminate from the apical surface and move to the SVZ (outer SVZ for bRG). Whereas bRG inherit the basal process and retain the stemness features of aRG, IP/BP lose both the apical and basal processes, are fate committed, and divide a limited number of rounds to increase the final output of neurons. IP/BP and neurons are produced by asymmetric/differentiative division, and settle at more dorsal positions than their progenitors. Differentiation of aRG has been correlated with an orientation of mitotic spindle that is perpendicular (cleavage plane parallel) to the ventricular surface (Chenn and McConnell, 1995; Shitamukai et al., 2011), even though this is still a matter of controversy, as some authors have reported that the spindle orientation and fate decision and not strictly correlated, and that the cleavage plane of aRG division is always perpendicular to the ventricular surface irrespective to whether they self-renew or differentiate (Noctor et al., 2008). Nonetheless, upon differentiation, cellular components and/or molecules involved in fate determination such as the apical/basal process, mother centriole, cilia remnants, or Numb/Notch are unequally inherited between the daughter cells. The type and timing of cell division are key determinants of cortical neurogenesis and both apical–basal polarity and planar polarity have been involved.

### Role of the Par3-Par6-aPKC System During Corticogenesis

The Pars are scaffold proteins able to bind to each other and interact with multiple cell polarity proteins (Figure 1A) (Wen and Zhang, 2018). Par3, Par6 and aPKC are interdependent for asymmetric localization in the cell cortex (Hong, 2018). Par3-Par6-aPKC complex has a key role in establishing apical–basal polarity by mediating the first steps of AJCs formation in nascent cell–cell contact sites (Suzuki and Ohno, 2006). Par proteins participate to many cellular processes regulating cell proliferation, migration and differentiation (Rodriguez-Boulan and Macara, 2014). Pard3, Pard6, and aPKC are expressed at the apical surface of the mouse VZ during neurogenesis (Costa et al., 2008). Mammalian homologs of *Drosophila* Par3 are Pard3 and Pard3b (Hapak et al., 2018). Pard3 colocalizes with N-cadherin, β-catenin, and especially with Zonula Occludens-1 (ZO-1) in aRG AJCs (Bultje et al., 2009); and is an important regulator of aRG growth, proliferation and differentiation (Bultje et al., 2009; Liu et al., 2018). By interacting with Hippo and Notch pathways, Pard3 regulates the balance between proliferation and differentiation in temporal manner through mouse cortical neurogenesis. Pard3 removal promotes aRG symmetric division at the expense of early born neurons. This results in excessive production of progenitors which are ectopically distributed throughout the cortical wall (Liu et al., 2018). This is believed to be caused by the inability of the neuronal progenitors to establish normal AJCs that hold them in the VZ (Bultje et al., 2009; Liu et al., 2018). At later stages of neurogenesis, Pard3 depletion promotes aRG symmetric differentiation and results in overproduction of late born neurons (Costa et al., 2008; Bultje et al., 2009; Liu et al., 2018). This leads to severe cortical malformation associated with abnormal lamination, increase in cortical size, and ribbon-like heterotopia (Liu et al., 2018).

Pard6a, Pard6b, and Pard6g are the mammalian homologs of *Drosophila Par6* (Hapak et al., 2018). Pard6 proteins have distinct expression patterns and functional properties in mammalian epithelial cells (Gao and Macara, 2004). The function of Pard6 in neuroepithelium polarization and cortical neurogenesis was assessed *in vitro* and/or by *in utero* electroporation, and no data from knockout mice is available to substantiate Pard6 functions *in vivo*. Pard6 overexpression in neural progenitors promotes symmetric divisions leading to the accumulation of neural progenitors in VZ, but not in SVZ (Costa et al., 2008). Intriguingly, Pard6 overexpression and Pard3 loss-of-function produce opposite proliferation phenotypes in neuronal progenitors *in vitro*, but similar phenotypes *in vivo* (Costa et al., 2008; Liu et al., 2018).

Mammalian homologs for *Drosophila* aPKC are Prkci and Prkcz. The *Prkci* gene encodes aPKCλ. *Prkcz* encodes two independent transcripts, aPKCζ and PKMζ (Hapak et al., 2018). aPKCλ and PKMζ are highly expressed in the brain, while aPKCζ is not (Soloff et al., 2004; Lee et al., 2013). Functional studies in mice showed that aPKCλ deficiency leads to early embryonic lethality, and that aPKCζ and PKMζ are dispensable for brain development. *Prkcz* knock-out mice are viable, and have no obvious defects (Soloff et al., 2004; Lee et al., 2013). aPKCλ has also been reported to be dispensable for establishment of apical–basal polarity in early embryos (Seidl et al., 2013). aPKCλ deficiency impairs tight junction protein ZO-1 localization and leads to disorganized epithelial structures in embryos (Seidl et al., 2013). Therefore, aPKCζ and PKMζ are not critical for establishing apical–basal polarity in brain neuroepithelium, even though brain specific conditional knock-out mice and close scrutiny would be required to reveal possible functions of aPKCλ during later embryonic brain development.

### Crumbs-Pals1-PatJ

*Drosophila* Crumbs is a transmembrane protein with three mammalian homologs, Crb1, Crb2, and Crb3 which are all expressed in brain (den Hollander et al., 2002; Makarova et al., 2003; Assemat et al., 2008). Crb1 and Crb3 knockout mice have not revealed any defects in developing cerebral cortex (Chou et al., 2018). However, Crb2 deficiency results in disorganized neuroepithelium, premature neuronal differentiation, and defective cortical lamination (Xiao et al., 2011; Dudok et al., 2016). These defects, however, are mild and transient suggesting that Crb2 is not critical for the development of cerebral cortex (Dudok et al., 2016).

Pals1 [also known as membrane protein, palmitoylated 5 (Mpp5) or MAGUK p55 subfamily member 5] is a mammalian protein homolog of *Drosophila* Stardust. Pals1 serves as an adaptor protein mediating interactions between Crbs and Patj. It has been suggested that Pals1 links Crumbs-Pals1-PatjJ and Par3-Par6-aPKC apical complexes (Figure 1A). Pals1 is expressed in apical membrane of the mouse brain progenitors (Kim et al., 2010). Unlike Crbs, Pals1 depletion results in lack of cortical neurons (Kim et al., 2010). Pals1 deficiency causes premature cell cycle exit of the VZ progenitors which results in severe reduction in their numbers. In addition, absence of Pals1 promotes cell fate switch from progenitors to neurons inducing an excessive production of early postmitotic neurons. The majority of these neurons die later causing a reduction in the size of the cerebral cortex (Kim et al., 2010).

*Drosophila* DPatj protein have two mammalian homologs Patj and Mupp1 (also known as Mpdz). Patj interacts with Crb3 and Pals1 in the apical domain linking them to TJs (Assemat et al., 2008). Mupp1/Mpdz acts as a scaffold protein to TJs components, e.g., JAMs and claudins (Assemat et al., 2008). Even though Mupp1/Mpdz deficiency does not seem to affect TJs formation and apical–basal polarity in mouse neuroepithelium (Feldner et al., 2017), it disrupts the integrity of ependymal cells and results in hydrocephalus (Feldner et al., 2017; Saugier-Veber et al., 2017).

### Basolateral Scribble-Lgl-Dlg Complex

Scribble, Lgl and Dlg show strong genetic interaction and share many phenotypic similarities in *Drosophila* (Bilder and Perrimon, 2000; Bilder et al., 2000; Bonello and Peifer, 2019). However, evidence for direct molecular interactions between these proteins are insufficient to enlighten the functions they carry out together and/or separately (Campanale et al., 2017; Bonello and Peifer, 2019). Scribble, Lgl and Dlg localize to the apico-basolateral boundary in epithelial cells (Figure 1A). Loss of Scribble, Lgl, or Dlg results in mislocalization of the apical proteins and AJCs to the basolateral domains. This leads to defects in cell architecture and impairs apical–basal polarity (Bilder and Perrimon, 2000; Bilder et al., 2000). Mouse homolog Scribble1 (Scrb1) deficiency results in severe neural tube defects and neonatal lethality (Murdoch, 2003; Kharfallah et al., 2017). Interestingly, Scribble1 interacts with planar cell polarity proteins in *C. elegans* and mouse (Montcouquiol et al., 2003, 2006; Murdoch, 2003; Phillips et al., 2007; Hoffmann et al., 2010). Loss of Scrb1 or PCP proteins Vangl2, Celsr1, and Fzd3/Fzd6 all result in similar neural tube defects (Kibar et al., 2001; Murdoch, 2003; Tissir and Goffinet, 2013b). Scrb1 affects Vangl2 localization directly and/or indirectly through apical protein Pard3 (Kallay et al., 2006; Montcouquiol et al., 2006; Kharfallah et al., 2017). Therefore, in addition to its role in apical–basal polarity, Scribble has a functional role in planar cell polarity.

Mice have two *Drosophila* Lgl homologs, Llgl1 and Llgl2, of which only Llgl1 is expressed in developing brain (Klezovitch et al., 2004; Sripathy et al., 2011). Llgl1 competes with Pard3 to balance TJs formation in epithelial cells by directly forming complexes with Pard6-aPKC (Figure 1A) (Yamanaka et al., 2003). In addition, Llgl1 promotes N-cadherin endocytosis in basolateral cell membrane which is inhibited by aPKC mediated phosphorylation of Llgl1 in apicolateral membrane. This polarized regulation of N-cadherin in apical–basal axis is essential to maintain neuroepithelial integrity (Klezovitch et al., 2004; Jossin et al., 2017). In line with this, Llgl1 deficiency in mouse results in mispolarization of N-Cadherin based AJs which leads to the disrupted neuroepithelium (Klezovitch et al., 2004; Jossin et al., 2017). Lgl/Llgl1 depletion in neural progenitors results in elevated aPKC activity which impairs Numb mediated regulation of Notch signaling (Haenfler et al., 2012). This might lead to sustained proliferation of neural progenitors and their failure to differentiate, a finding that has been reported both in *Drosophila* and mouse (Klezovitch et al., 2004; Haenfler et al., 2012). Lgl/Llgl1 deficiency severely alters proliferation and differentiation leading to periventricular heterotopia (Klezovitch et al., 2004; Jossin et al., 2017).

Mammalian homologs of *Drosophila* Dlg, Dlg1 (SAP97), Dlg2 (PSD-93), Dlg3 (SAP102), and Dlg4 (PSD-95/SAP90), belong to a subfamily of membrane-associated guanylate kinases (MAGUKs) (Stephens et al., 2018; Ye et al., 2018). Dlgs share three protein domains, namely, PSD-95/Dlg/ZO-1 (PDZ), Src homology domain-3 (SH3) and guanylate kinase like (GUK) domain, through which they interact with numerous proteins involved in different cellular functions (Stephens et al., 2018). *Dlg* mice do not exhibit any abnormalities or absence of apical–basal polarity in forebrain neuroepithelium (Migaud et al., 1998; Caruana and Bernstein, 2001; McGee et al., 2001; Cuthbert et al., 2007; Van Campenhout et al., 2011), which could be due to the functional redundancy of Dlgs (Caruana and Bernstein, 2001; McGee et al., 2001). Interestingly, like Scribble, Dlgs have been reported to interact with planar cell polarity protein Vangl2 (Yoshioka et al., 2013; Stephens et al., 2018). Dlg1 depletion share phenotypic features with PCP-deficient mice, including defects in neural tube closure (Caruana and Bernstein, 2001; Rivera et al., 2013).

### PCP and Cortical Neurogenesis

Planar cell polarity has a documented role in the oriented cell division (OCD) in *Drosophila* (Bellaiche et al., 2001; Segalen and Bellaiche, 2009) and zebrafish (Gong et al., 2004; Segalen et al., 2010), and PCP components are expressed in mammalian cortical progenitors (Tissir et al., 2002a,b; Tissir and Goffinet, 2006; Ishiuchi et al., 2009). Surprisingly, the relationship between PCP, OCD, and fate determination of cortical progenitors has been slow to emerge. Fat4 and Dchs1 colocalize with the Crumbs-Pals1-Patj1 complex at cell–cell contact site. Fat4 and Dchs1 interact in a heterophilic manner, affecting their respective levels, and organizing, together with Pals1, the apical membrane architecture. However, this presumed role awaits further validation as, to the best of our knowledge, no functional analysis was performed to assess this *in vivo*. Mice carrying the *Looptail* (*Lp*) mutation in Vangl2 (this allele has a dominant negative activity) (Yin et al., 2012) exhibit precocious differentiation of cortical progenitors (Lake and Sokol, 2009). *Lp/Lp* neural progenitors showed premature differentiation, a loss of asymmetric distribution of Leu-Gly-Asn-enriched protein (LGN)/Partner of Inscuteable (Pins), a regulator of mitotic spindle orientation. This was accompanied by an increase in the number of vertical cleavage planes typically associated with equal daughter cell identities, suggesting that Vangl2 functions to maintain cortical progenitors (Lake and Sokol, 2009). Celsr1 is also involved in cortical neurogenesis where is plays an opposite role to that of Vangl2. Celsr1-deficient cortical progenitors undergo more proliferative divisions, expanding the pool of progenitors at the expense of IP/BP and neuron production. This results in a reduced number of cortical neurons, abnormal brain architecture (thicker VZ/SVZ, and thinner upper layers of the neocortex), microcephaly, and behavioral impairment. *Celsr1* cortex-specific mutant mice are hyperactive and exhibit deficits in attention, social interactions, and learning and memory (Boucherie et al., 2018). This combination of phenotypes is evocative of human neurodevelopmental disorders where hyperactivity disorder/attention deficit and autism spectrum disorder frequently co-occur. In absence of Celsr3 or Fzd3, it is the gliogenic switch that is affected. Neurogenesis is delayed while gliogenesis is decreased. The phenotype is not due to gene function in cortical progenitors, but rather in immature cortical neurons that fail to upregulate expression of Jag1 in response to cortical Wnt7, resulting in reduced activation of Notch signaling in cortical progenitors. In fact, Celsr3 and Fzd3 enable immature neurons to respond to Wnt7, upregulate Jag1 and hence facilitate feedback signals that tune cortical progenitor fate decisions via Notch activation (Wang W. et al., 2016).

## Apical Basal Polarity and Radial Migration of Cortical Neurons

Apicobasal polarity is inherited by nascent postmitotic neurons as front-to-rear polarity (Kon et al., 2017). Excitatory neurons of the cerebral cortex are generated in VZ and SVZ from where they migrate radially to the appropriate cortical layers following an inside-out sequence. During radial migration, these cells dynamically change their migration mode from somal translocation (Figure 1B), to a multi-phase migration that comprises multipolar migration, glia-guided locomotion (locomotion), and final somal translocation (Figure 1C). At early stages of cortical development (i.e., E10–E13), a majority of the ventricular zone (VZ) born neurons migrate using somal translocation, until the intermediate zone (IZ) and cortical plate (CP) form (Nadarajah et al., 2001; Cooper, 2013). These translocating neurons inherit the basal process from their radial glial progenitors and remain attached to the pia (Miyata et al., 2001), while the apical process detaches from VZ surface (Figure 1B). At later stages (E14–E18), migrating cells adopt a multipolar shape as they move from the VZ and SVZ to the lower IZ (Tabata and Nakajima, 2003; Noctor et al., 2004). In IZ, multipolar cells extend and retract numerous processes in various directions. They also frequently change their migration direction both in tangential and radial axis, although the net movement remains toward CP (Tabata and Nakajima, 2003; Noctor et al., 2004; Tabata et al., 2009; Kon et al., 2017). When multipolar neurons reach the upper IZ,their cellular morphology changes from multipolar to bipolar shape (Ohshima et al., 2007; Cooper, 2014), a transition accompanied by a shift from multipolar migration to locomotion (Tabata and Nakajima, 2003; Noctor et al., 2004). Locomoting neurons move along radial glia fibers and are not attached to the pial surface (Nadarajah et al., 2001). They move in upper IZ and CP by cycles of saltatory migration. Each cycle begins by extending a leading process toward the marginal zone (MZ). This is followed by movement of the centrosome and Golgi apparatus to the proximal part of leading process. Finally, nucleus translocate into the leading process as trailing process detaches from radial glia leading to a net movement of the neuron (Miyata et al., 2001; Noctor et al., 2004; Solecki et al., 2004; Tsai et al., 2007). Nucleus, centrosome, and Golgi positioning and movement are connected to each other, and to actin and microtubule (MT) cytoskeleton (Solecki et al., 2004; Bellion et al., 2005; Barker et al., 2016). In the final phase of radial migration, neurons detach from radial glia as their leading processes contact the MZ and cell soma translocate to their ultimate position in CP (Nadarajah et al., 2001; Sekine et al., 2014) (Figure 1C). This last stage is called “terminal translocation” and its importance remains unclear.

### Neural Delamination and Initiation of Migration

Delamination, that is detachment of cells from neuroepithelium, is a hallmark for the migration initiation from VZ and/or SVZ toward the CP. Delamination begins with disassembly of apical AJCs and centrosome-cilium complex (basal body), characterized by Foxp2- and Foxp4-mediated downregulation of N-cadherin in neural progenitors (Rousso et al., 2012). N-Cadherin downregulation is necessary for the centrosome release from basal body and disassembly of N-cadherin based AJCs (Rousso et al., 2012; Das and Storey, 2014). N-cadherin downregulation is also required for the acto-myosin mediated abscission and shedding of apical membrane (Das and Storey, 2014; Kasioulis et al., 2017). Delamination requires basal translocation of centrosome which is regulated by the interaction between actin and microtubule cytoskeleton (Kasioulis et al., 2017). Delamination can be alternatively achieved through asymmetric division of apical progenitors. Depending on the cleavage plane, apical, and basal domains can be inherited asymmetrically between daughter cells. Therefore, postmitotic cells may inherit only the basal domain of their parent cell and lose apical AJCs and connection to the apical belt in the process (Taverna et al., 2014; Wilsch-Brauninger et al., 2016).

Pard6 overexpression causes accumulation of neural progenitors in VZ, and it has been suggested that this is due to defective initiation of radial migration (Solecki et al., 2004; Costa et al., 2008). Pard6 also regulates neuronal migration *in vitro* through centrosome repositioning and nuclear translocation (Solecki et al., 2004; Costa et al., 2008). Patj may also be important for neuronal migration as it regulates the localization of Pard3 and aPKC, as well as the reorientation of MT and centrosome in the leading process (Shin et al., 2007; Du et al., 2010). The transcription factor insulinoma-associated 1- (INSM1) mediated downregulation of pleckstrin homology domain containing, family A member 7 (Plekha7) is required for the delamination of the apical progenitors (Farkas et al., 2008; Tavano et al., 2018). Plekha7 is an AJ protein that interacts with nectin–afadin complex to form and stabilize AJs. Thus, its downregulation might induce AJC disassembly leading to delamination of progenitors (Farkas et al., 2008; Meng et al., 2008; Kurita et al., 2013; Tavano et al., 2018). In addition, Filamin A (Flna), an actin binding protein, is suggested to affect initiation of migration of postmitotic neurons in two ways. First, Flna degradation by the Filamin A interacting protein 1 (Filip1) is required for the formation and maintenance of the bipolar shape through changing actin dynamics (Nagano et al., 2002; Nagano et al., 2004). Second, Flna participates to trafficking of adhesion proteins through brefeldin A inhibited guanine exchange factor (BIG2), which may cause alterations in AJCs assembly/disassembly (Zhang et al., 2013). Based on these studies, it is evident that apical AJCs disassembly is a major factor for delamination. Defects in delamination and initiation of the radial migration results in periventricular heterotopia (PVH) both in mice and humans (Kasioulis et al., 2017). PVH is a common cortical migration defect and has at least 15 subtypes (Battaglia et al., 2006; Parrini et al., 2006). PVH is characterized by ectopically located nodules of neurons along the lateral ventricle walls (Lian and Sheen, 2015; Romero et al., 2018). In humans, PVH can cause epileptic seizures, dyslexia and mild psychiatric disorders (d’Orsi et al., 2004; Battaglia et al., 2006; Lian and Sheen, 2015). It can also occur concomitantly with other conditions and syndromes (Sheen and Walsh, 2005; Leibovitz et al., 2018). PVH comprises up to 31% of the neuronal migration related cortical malformation (Broix et al., 2016; Romero et al., 2018). Two most familiar genes associated with PVH are X-linked *FLNA* and *ARFGEF2* which encodes BIG2 protein (Sheen et al., 2001; Sheen et al., 2004). X-linked *FLNA* mutations account for about 50% of the PVHs, which are primarily present in females (Fox et al., 1998; Parrini et al., 2006; Broix et al., 2016). *ARFGEF2* mutations produce a rare form of PVH linked with severe developmental delay and microcephaly (Lian and Sheen, 2015; Yilmaz et al., 2016).

### Multipolar Migration

Multipolar migration starts when postmitotic cells detach from aRG and undergo the first polarity transition from bipolar to multipolar state (Figure 1C). The detachment and the first polarity transition are not well understood (Cooper, 2014; Kon et al., 2017). However, some studies suggest that defects in glial-guided locomotion lead to accumulation of postmitotic neurons in VZ-SVZ/IZ border. For instance, WNT/β-catenin signaling and gap junction subunits Connexins 26/43 have been reported to affect the morphology and adhesion of postmitotic neurons to radial glia in SVZ/IZ (Elias et al., 2007; Boitard et al., 2015; Bocchi et al., 2017; Ji et al., 2017). During the multipolar migration from lower IZ to the upper IZ, cells do not exhibit any clear sign of polarity (Sakakibara et al., 2014). The second transition from multipolar to bipolar state is better understood and several genes/signaling pathways have been implicated. Defective multipolar–bipolar transition often leads to accumulation of cells in the IZ. Key targets of signaling pathways involved in the multipolar–bipolar transition are the small GTPases (Ohtaka-Maruyama and Okado, 2015). RhoA is an important regulator of the cytoskeleton dynamics and its activity is tightly regulated by several effectors. Proneural transcription factors Neurogenin 2 (Neurog2) and Achaete-scute homolog 1 (Ascl1) induce expression of Rho GTPases Rnd2 and Rnd3 in migrating neurons. In turn, these GTPases inhibit RhoA activity and promote multipolar–bipolar transition (Heng et al., 2008; Pacary et al., 2011). In addition, Neurog2-Rnd2 pathway has an interactive feedback loop with zinc finger transcription factor RP58 (Zfp238), a downstream target of Neurog2 that represses transcription of both Neurog2 and Rnd2 (Ohtaka-Maruyama et al., 2013; Heng et al., 2015). Ascl1-Rnd3 pathway is connected to extracellular signaling by a member of Semaphorin-Plexin pathway, Plexin B2 receptor, which interacts with Rnd3 to regulate RhoA activity (Azzarelli et al., 2014). RhoA activity is also regulated by cyclin dependent kinase 5 (Cdk5) acting through p27Kip1 and/or serine/threonine kinase Mst3 to decrease RhoA activity and induce multipolar–bipolar transition (Kawauchi et al., 2006; Nguyen et al., 2006; Tang et al., 2014). In addition to p27Kip1 and Mst3, Cdk5 phosphorylates many proteins involved in multipolar migration, including MT-associated proteins Dcx, Ndel1, Crmp2, and actin-associated proteins Drebrin (Dbn) and p35 (Chae et al., 1997; Niethammer et al., 2000; Tanaka et al., 2004; Uchida et al., 2005; Cooper, 2014; Ohshima, 2014; Tanabe et al., 2014; Ohtaka-Maruyama and Okado, 2015; Shah and Rossie, 2018). In addition to Plexin B2, Netrin receptors Unc5D and DCC participate in multipolar migration. Unc5D expression increases in the beginning of the multipolar migration and its repression by transcription factor FoxG1 is required for the multipolar–bipolar transition (Sasaki et al., 2008; Miyoshi and Fishell, 2012). DCC has been reported to interact with Disabled-1 (Dab1, a Reelin adaptor protein) independently from Reelin signaling to enhance multipolar–bipolar transition (Zhang et al., 2018). The Reelin signaling component, N-Cadherin is a key adhesion protein during multipolar migration (Kawauchi et al., 2010; Jossin and Cooper, 2011; Shikanai et al., 2011; Matsunaga et al., 2017; Horn et al., 2018). N-cadherin localization and levels at the plasma membrane affect process formation, multipolar–bipolar transition, and entry of neurons in CP. Reelin, fragile X mental retardation protein (FMRP), adaptor protein Drebrin-like (Dbnl), and CDK5 regulate N-cadherin levels in multipolar neurons (Jossin and Cooper, 2011; La Fata et al., 2014; Ye et al., 2014; Inoue et al., 2019). In addition, N-Cadherin expression and subcellular distribution are mediated by several small GTPases (Kawauchi et al., 2010; Jossin and Cooper, 2011; Kawauchi, 2011; Ye et al., 2014; Hor and Goh, 2018). Recently it was also reported that synaptic transmission from early subplate neurons enhances multipolar–bipolar transition (Ohtaka-Maruyama et al., 2018).

### Glial-Guided Locomotion

The glia-guided locomotion begins when migrating cells adhere to radial glia in the upper IZ, and ends when the leading edge reach the pial surface and cell body the primitive cortical zone (Figure 1C) (Cooper, 2013). During locomotion, neurons extend their leading process toward the CP and form a dilation (swelling) to the proximal part of the leading process. Cell organelles move to this dilation in sequential manner. The centrosome and Golgi apparatus moves first, followed by the elongated nucleus. The sequential movement of the cell organelles is repeated in cycles which produce so-called “two stroke cycle” and distinctive saltatory movement of the cell soma (Nadarajah et al., 2001). Adhesion to aRG has a significant role in providing the necessary traction required for the cell and cell organelles movement. New adhesions are generated in the leading process as it is extending, adhesions beneath the cell soma are relatively stable, and those in trailing process are constantly removed which enables its retraction (Schaar and McConnell, 2005; Shieh et al., 2011; Solecki, 2012; Kawauchi, 2015; Bertipaglia et al., 2018). Therefore, the adhesion protein trafficking is one of the major regulators of locomotion together with translocation of cell organelles (Rakic, 1972; Gregory et al., 1988; Kawauchi et al., 2010; Wilson et al., 2010; Shieh et al., 2011; Solecki, 2012). For instance, endocytosis and adhesion receptor recycling promoting adhesion between neurons and glia has been reported for N-cadherin, astrotactin (Astn) 1 & 2, and integrins (Anton et al., 1996; Dulabon et al., 2000; Adams et al., 2002; Kawauchi et al., 2010; Wilson et al., 2010). In addition, N-Cadherin and Astn1 direct interaction, in *cis* and *trans*, promotes locomotion in cerebellum (Horn et al., 2018). Cx26 and Cx43 localize to the neuron-glia adhesions and enable stabilization of the leading process, required for the somal translocation (Elias et al., 2007). Furthermore, Cx26 mediated adhesion and assembly at the neuron-glia contact sites requires the focal adhesion kinase (FAK) and paxillin (Valiente et al., 2011; Rashid et al., 2017). Pard3 mediated recruitment of junctional adhesion molecule C (Jam3) to the neuron-glia contact sites promotes adhesion between migrating neurons and RG fibers (Famulski et al., 2010).

As mentioned above, adhesion protein trafficking provides the necessary traction for the forward movement of the cell. The required force to pull and push cell organelles is mediated by the intracellular MTs and actomyosin (Rivas and Hatten, 1995; Bellion et al., 2005). Formation of the leading process dilation precedes cell organelle movement and requires regulation of both MT and F-actin dynamics (Schaar and McConnell, 2005; Nishimura et al., 2017). Rac1 and its binding partner Posh (aka Sh3rf1: SH3 domain containing ring finger 1) regulate formation of the dilation by mediating F-actin assembly (Yang et al., 2012). On the other hand, CDK5 and its substrates Dcx and p27Kip1 regulate the formation of dilation through modulating MTs and endocytic trafficking (Nishimura et al., 2014). Defects in the formation of leading process dilation have been suggested to perturb migration by affecting centrosome/nucleus movement into the dilation, (Yang et al., 2012; Nishimura et al., 2014). Centrosome movement into the dilation is also regulated by dynein and its regulator Lissencephaly 1 (Lis1). It has been proposed that at the dilation, cell cortex connected dynein/Lis1 complexes generate forces (mediated by the MT) that pull the centrosome toward the dilation (Tsai and Gleeson, 2005; Tsai et al., 2007). After centrosome movement, the nucleus is transported into the dilation. Nucleus is surrounded by specific MT structure (named MT-cage or fork) which extends to the centrosome (Rivas and Hatten, 1995; Xie et al., 2003). Linker of nucleoskeleton and cytoskeleton (LINC) protein complexes couples nucleus to the surrounding MT-cage. The LINC protein complexes in mice include Sun1/2 and KASH domain proteins Syne/(Nesprin) 1/2/3 (Barker et al., 2016; Lee and Burke, 2018). Sun1/2 and Syne2 form complexes and mediate coupling of the nucleus and centrosome during migration (Zhang et al., 2009). Sun1/2 recruit Syne2 to the nuclear envelop (NE), as Syne2 is able to interact with cytoplasmic kinesins and dynein/Lis1. It has been proposed that Sun1/2 anchor to the inner NE and form complexes with Syne1/2 in outer NE. In turn, Syne1/2 interact with cytoplasmic dynein/Lis1 complex to pull the nucleus toward the centrosome and with kinesin to push nucleus away from the centrosome (Zhang et al., 2009). In conclusion, Sun1/2 and Syne1/2 participate in mediating MT-driving force to the nucleus via dynein/Lis1 and kinesin complexes. Loss of LINC protein complexes results in defective nuclear movement together with migration failure in cerebral cortex (Zhang et al., 2009; Lombardi et al., 2011). In addition to the movement of centrosome and nucleus mediated by the dynein/Lis1/MT pathway, actomyosin contraction generate pulling force to the centrosome from the leading process, and pushing force to the nucleus from the trailing process moving them toward the dilation (Bellion et al., 2005; Schaar and McConnell, 2005; Solecki et al., 2009).

Genetic mutations associated with migration disorders are involved in cortical malformations such as PVH (discussed earlier in section “Neural Delamination and Initiation of Migration”), lissencephalies (“smooth brain”) and tubulinopathies. Lissencephalies include lissencephaly and cobblestone lissencephaly (Barkovich et al., 2012; Devisme et al., 2012; Romero et al., 2018). Lissencephaly comprises agyria (smooth and disorganized without gyri), pachygyria (abnormal gyri), and ectopic neurons forming subcortical band heterotopia or double cortex (Romero et al., 2018). Mutations in 20 genes have been associated with lissencephaly, but the most important ones are *LIS1, DCX, TUBA1A*, and *DYNC1H1* that account for nearly 71% of the patients (Di Donato et al., 2018). Lissencephaly causes feeding difficulties, hypotonia, epileptic seizures, and intellectual disability (Guerrini and Dobyns, 2014; Romero et al., 2018). Cobblestone lissencephaly is characterized by overmigration of neurons (Devisme et al., 2012), and disruption of the basement membrane (Nakano et al., 1996; Satz et al., 2010). Tubulinopathies are caused by mutations in genes encoding tubulin isoforms. Seven genes have been associated with tubulinopathies, namely, *TUBA1A, TUBB2A, TUBB2B, TUBB3B, TUBB4A, TUBB* (*TUBB5*), and *TUBG1* (Bahi-Buisson et al., 2014; Romaniello et al., 2018; Romero et al., 2018). These genes encode α- and β-tubulin isoforms which are the building blocks of MT. Tubulinopathies cause epileptic seizures, intellectual disability, motor delays, and perinatal lethality in the most severe cases (Bahi-Buisson et al., 2014; Sweet et al., 2017; Romaniello et al., 2018).

## Axon and Dendrites Specification in the Cerebral Cortex

### Cell Intrinsic Mechanisms

**FIGURE 2:**
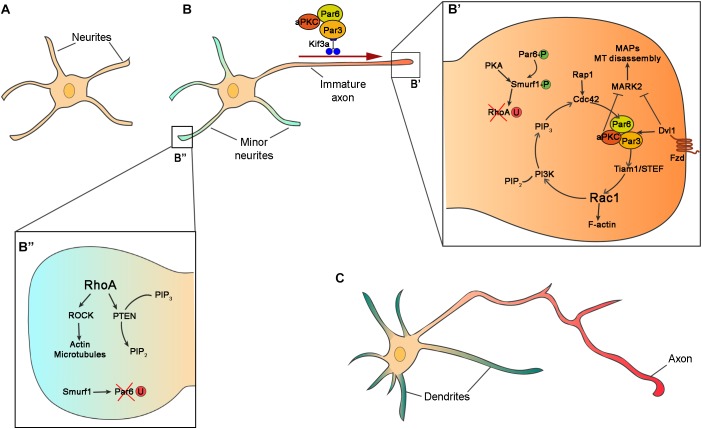
Intrinsic mechanisms for neuronal polarization *in vitro*. **(A)** Schematic representation of hippocampal neurons after 10 h *in vitro* with similar neurites in term of length and cytoskeleton architecture. **(B)** In the second stage of polarization, hippocampal neuron at stage of axon/dendrite specification. One of the neurites grows faster and becomes the axon. **(B’,B”)** Zooms in the boxed regions of **(B)** showing intracellular signaling of Par complex (Par3, Par6, and aPKC), and other polarity effectors in axon **(B’)** and dendrite **(B”)** specification. In the prospective axon, Par6 and Smurf1 are phosphorylated (Par6-P and Smurf1-P) and RhoA is degraded by ubiquitination (RhoA-U), while in the minor neurites Par6 is ubiquitinated (Par6-U). **(C)** Polarized neurons exhibit several dendrites (green) and one axon (red).

For many years, the primary culture of hippocampal neurons was the experimental system of choice to study cell-autonomous factors for neuronal polarization and specification of the axon and dendrites. Dissociated neurons cultured *in vitro* are initially round-shaped immediately after plating. Ten hours later, they undergo cytoskeletal rearrangement and develop neurites. At this stage, neurons are multipolar with similar neurites in term of length, and F-actin and MT distribution (Figure 2A). From this stage onwards, one neurite will break the symmetry, grow fasters than the others, and develops as an axon, whereas the others form the dendrites (Figure 2B,C). The length of the neurite is a key factor for axon specification, and cytoskeleton dynamic is the main player for neurite outgrowth (Lamoureux et al., 2002; Witte et al., 2008; Yamamoto et al., 2012). MT orientation in axon and dendrites is also polarized (Baas et al., 1988). While in the axon, all MT plus-ends of are oriented toward the growth cone (plus-end out), dendrites possess MT with both orientations, with plus-end and minus-end toward the dendritic tip (Kapitein and Hoogenraad, 2011; Yau et al., 2016; Park and Roll-Mecak, 2018). This asymmetric distribution influences the dynamic instability of the MT since the plus-ends microtubules at the tip of the axon are more susceptible to growth and collapses depending on changes in the extracellular environment (Kollins et al., 2009; Akhmanova and Hoogenraad, 2015). Actin, MT, and polarity proteins are, therefore, key to axon/dendrite specification since they regulate the differences in cytoskeleton architecture.

The protein complex formed by Par3-Par6-aPKC is one of the best-studied signaling molecules in cell polarization. In cultured hippocampal cells, Par complex proteins accumulate at the distal tip of the prospective axon and are implicated in axon specification (Shi et al., 2003; Nishimura et al., 2004; Schwamborn and Puschel, 2004; Shi et al., 2004) (Figure 2B’). Par3 and Par6 are scaffold proteins that regulate MT stability promoting neurite elongation (Watts et al., 1996; Chen et al., 2013). Par6 interacts with Par3 to bind aPKC (Joberty et al., 2000; Johansson et al., 2000; Qiu et al., 2000). Par3-Par6-aPKC complex associates with the guanine exchange factor (GEF) Tiam1/STEF regulating the activity of the small GTPase Rac1 (Lin et al., 2000; Mertens et al., 2005; Nishimura et al., 2005). The activation of Tiam1/STEF increases the activity of Rac1 at the tip of the axon stimulating the activation of Phosphoinositide 3-kinases (PI3Ks). PI3K produces phosphatidylinositol (3,4,5)-trisphosphate (PIP3), which accumulates in the axon at the expense of PIP2. The activity of PI3K is counteracted by the protein phosphatases PTEN (phosphatase and tensin homolog deleted on chromosome 10) (Maehama and Dixon, 1998). PTEN revert PI3K activity dephosphorylating PIP3 into PIP2 and therefore regulates the spatial and temporal concentration of PIP3 (Carracedo and Pandolfi, 2008; Park et al., 2008). PIP3, in turn, activates another small GTPase, Cdc42 (Etienne-Manneville and Hall, 2001; Yamanaka et al., 2001; Nishimura et al., 2005). Cdc42 regulates cytoskeleton dynamics (Hall, 1993) and interacts with Par6, closing the loop in positive feedback (Schwamborn and Puschel, 2004) (Figure 2B’).

Par protein complex also interacts with other effectors to stimulate neurite outgrowth and axon specification. aPKC in association with Par3 and Par6 negatively regulates Mark2 in the axons (Chen et al., 2006). Mark2 (also called Par-1), an MT-binding protein, activates MT associated proteins (MAPs) such as Tau-1, Map2/4, and Dcx, promoting MT disassembly (Drewes et al., 1997; Matenia and Mandelkow, 2009; Reiner and Sapir, 2014). The inhibition of Mark2 in hippocampal neurons results in the formation of multiple axons, whereas overexpression prevents neuronal polarization (Chen et al., 2006; Wu et al., 2011). Hence, the inactivation of Mark2 by aPKC promotes neurite elongation and axon specification (Chen et al., 2006). Phosphorylated Par6 recruits the ubiquitin ligase Smurf-1 at the axon to degrade the small GTPase RhoA (Ozdamar et al., 2005; Wang H. R. et al., 2006; Cheng et al., 2011). RhoA is a negative regulator of neuronal polarity inhibiting neurite growth. Unlike other Rho GTPase proteins, RhoA is degraded in the growth cone by Smurf1 and it is not implicated in axon specification, but it has a role in dendritic morphogenesis (Lee et al., 2000). The effector of RhoA is the Rho-kinase (Rock) which regulates actin and microtubules dynamics through phosphorylation of MAPs, LIM motif-containing protein kinase 2 (Limk2), and myosin (Amano et al., 1996; Kimura et al., 1996; Maekawa et al., 1999; Amano et al., 2003; Arimura and Kaibuchi, 2005). RhoA also regulates the localization and activity of Pten, which in turn regulates the production of PIP3 and PIP2, and indirectly the activity of PI3K (Li et al., 2005). aPKC can interact with other cell polarity proteins. The intracellular mediator of Wnt signaling Disheveled 1 (Dvl1, shared by Wnt/beta catenin and Wnt/PCP), accumulates at the end of the growing axon where it forms a complex with Par3-Par6-aPKC. Dvl1 activates aPKC and inhibits Mark2, promoting neurite elongation. Overexpression of Dvl1 produces multiple axons and its downregulation prevents axon formation (Zhang et al., 2007; Vyas et al., 2013). The role of Dvl1 in axon specification is downstream Wnt non-canonical signaling and therefore independent of Gsk3β–β-catenin pathway (Zhang et al., 2007).

Par3, Par6, aPKC proteins are distributed at the end of the longest neurite. But, how these proteins are selectively sorted in one neurite? Different mechanisms are implicated in the accumulation of Par proteins at the tip of the axon: anterograde transport, degradation, and local activation.

Polarity effectors can be transported using motor proteins including myosin, dynein, and kinesin. Myosin transport proteins along the F-actin filaments, meanwhile dyneins and Kinesins use MT as a substrate for protein transport. The most studied cargo proteins implicated in neuronal polarization are the kinesins. The superfamily of motor proteins Kinesin (KIFs) is composed of 15 Kinesin families with different functions. Kinesin are subdivided into three types depending on the position of the motor domain. The motor domain of Kinesins can be localized in the N-terminus (N-Kinesins), in the middle (M-Kinesins) or in the C-terminus (C-Kinesins) (Hirokawa et al., 2009). The localization of the motor domain determines their function. N-Kinesins move toward the plus-end of the microtubules and are therefore implicated in the anterograde transport in the axon. Kinesins can transport different types of cargo such as synaptic proteins, mitochondria, proteins implicated in axon elongation and neuronal polarization (Namba et al., 2011; Xiao et al., 2016). The protein complex composed by Par3-Par6-aPKC is transported the distal tip of the prospective axon by the N-Kinesin Kif3a (Shi et al., 2003; Nishimura et al., 2004). Par3 interacts directly with Kif3a acting as adaptor and regulates the delivery of the Par complex (Funahashi et al., 2013). When Par3 is phosphorylated by extracellular signal-regulated kinase (Erk2), it loses affinity for Kif3a and unloads the Par complex.

The localization of Par6 in the axon is also regulated by ubiquitination in the rest of the neurites. Smurf1 is phosphorylated in the axon by PKA, upon BDNF signal, and degrades RhoA preventing its activation in the axon. However, the non-phosphorylated Smurf1 in the rest of the dendrites degrades Par6 and restricts its localization to the axon (Cheng et al., 2011). This mechanism allows smurf1 to switch its substrate preference to favor Par6 accumulation and RhoA degradation in the future axon, promoting axon development. Since the amount of proteins is limited in a cell, the accumulation of these factors in a neurite would make it less available elsewhere, reinforcing the extension of the axon at the expense of the growth of the other neurites. This positive loop is called local activation global inhibition and it is used to explain the accumulation of growth factors, as the Par complex, at the tip of the axon (Schelski and Bradke, 2017).

### Cell Extrinsic Mechanisms

*In vitro*, all neurites grow freely and the axon specializes stochastically. However, neurons face different constraints and scenarios *in vivo*. Interactions with other cells, with the extracellular matrix (ECM), and with secreted cues, will determine the specification of the leading and trailing process, and therefore, the axon and dendrites.

Multipolar cells in the lower portion of IZ are “unpolarized” neurons extending neurites in various directions (Tabata and Nakajima, 2003; Noctor et al., 2004; Tabata et al., 2009). These neurons are in vicinity of pioneer neurons situated in the preplate. The first neurite to contact pioneer neuron axons will grow faster and will become the axon of future CP neuron (Namba et al., 2014). This interaction involves the contactin2 protein (Tag1), a contact cell protein expressed by the pioneer neurons (Denaxa et al., 2001; Espinosa et al., 2009; Namba et al., 2014). It is not clear whether this interaction facilitates the mechanical tension of the neurite allowing it to grow faster or, activates intracellular signaling implicated in neuronal polarization. This model of neuronal polarization *in vivo* is called “Touch and go” by the authors and predicts the specification of the axon precedes the bipolar transition (Denaxa et al., 2001; Espinosa et al., 2009; Namba et al., 2014) (Figure 1D). The second mechanism for axon specification rely on the contact between the neuron and radial glia. Kaibuchi and colleagues showed the specification of the axon occurs in the opposite neurite of the radial glia-contacting neurite. That means the contact between one neurite and the radial glial cell determines the neuronal polarization. This interaction is through N-Cadherin that regulates the polarized distribution of RhoA in dendrites and Rac1 in the prospective axon (Xu et al., 2015) (Figure 1D). In this model, bipolar transition precedes axon specification (Calderon de Anda et al., 2008).

Other factors of neuronal polarization *in vivo* include secreted factors such as Reelin (Jossin and Cooper, 2011), transforming growth factor b (Tgfb) (Yi et al., 2010) and semaphorins (Polleux et al., 1998; Shelly et al., 2011). Reelin is a glycoprotein secreted by Cajal-Retzius cells in the marginal zone (MZ) of the cortex and promotes the accumulation of N-Cadherin by the interaction of Rap1 and Reelin receptors (Jossin and Cooper, 2011). As we mentioned before, N-Cadherin accumulates in the neurite contacting the RG (the leading process) and governs axon specification (Xu et al., 2015). Therefore, reelin is an important factor for neuronal polarization *in vivo*. BDNF signaling, through the receptor TrKB, activates Lkb1 (also known as Stk11 or Par-4), which is important for axon specification in the cerebral cortex. The activated Lkb1 localizes at the end of the axon and phosphorylates Mark2 and Brsk2 (SAD-A) (Kishi et al., 2005; Barnes et al., 2007; Shelly et al., 2007). Both kinases regulate the activity of MT binding proteins and cytoskeleton dynamics. Interestingly, Knocking down Lkb1 in the cortex does not affect neuronal migration and dendritic formation but disrupts the formation of the axon (Kishi et al., 2005; Barnes et al., 2007; Shelly et al., 2007). A similar phenotype was found in cortical neurons lacking the TGF-b receptor 2, TgfbR2 (Yi et al., 2010). TgfbR2 phosphorylates Par6, activating Smurf1 and promoting RhoA degradation in the axon (Yi et al., 2010; Cheng et al., 2011). In the absence of TgfbR2, neurons display leading process toward the cortical plate but do not form axons (Yi et al., 2010). This is of special interest because it suggests that the axon specification is independent of bipolar polarization and neuronal migration.

Finally, Par3, partner of Par6 in axon specification in cultured hippocampal neurons, may also be implicated in neuronal polarization *in vivo*. Silencing Par3 in postmitotic neurons by *in utero* electroporation in the cerebral cortex, increases the number of multipolar cells in the lower IZ. Neurons fail to migrate to the cerebral cortex most probably due to defects in the multipolar to bipolar transition. Blocking the interaction of Par3 with the transporter molecule Kif3a yields the same phenotype (Funahashi et al., 2013). However, conditional ablation of Par3 in postmitotic cortical neurons (*Par3*^fl/fl^; *Nex-*Cre) does not affect neuronal migration or axon specification (Liu et al., 2018). This discrepancy maybe due to side effects of shRNAs *in utero* electroporation, or to compensatory mechanisms in mice.

## PCP Signaling and Directional Growth of Axons

Once neuronal migration is completed, neurons extend long-distance projections to connect with specific synaptic targets. In the cerebral cortex, excitatory pyramidal neurons are classified according to their molecular signature, projections, and laminar distribution. While the expression of a different combination of genes is essential for identity, and therefore for projections, the position in specific cortical layers seems to play a minor role. Mice with inverted layers or miss-located (ectopic) neurons maintain neuronal identity according to their birth-dating and extend the respective projections (Jensen and Killackey, 1984; Guy and Staiger, 2017). Pyramidal neurons in the cortex are categorized into intracortical, subcortical and subcerebral neurons. Intracortical projecting neurons are either ipsilateral (between the same hemisphere), or commissural neurons (contralateral hemisphere). The directional growth of axons is determined by the growth cone, a motile structure composed by F-actin rich lamellipodia and filopodia in the periphery domain, and MT extending in the central domain. The role of the growth cone is to explore the environment and guide the axon to the specific postsynaptic target. Hence, the growth cone membrane concentrates guidance receptors and cell adhesion proteins involved in the recognition of extracellular cues. The growth cone perceives and interprets attractive and repulsive guidance cues activating intracellular effectors that will result in axon extension and/or retraction. Axon guidance is also driven by guidepost cells, a cell population localized at intermediate target, that steer axon tracts toward final target territories (Squarzoni et al., 2015). Guidepost cells regulate axon formation by secreting guidance cues and setting cell–cell contact with the growth cone. Motor proteins (Kinesins, dyneins, and myosins) are key regulator of axonal transport and MT and actin organization in the growth cone (Kahn and Baas, 2016; Menon and Gupton, 2016).

The PCP proteins are not implicated in radial migration in the cortex, but they have key role in connectivity by regulating axon guidance and dendritic morphogenesis. Celsr1-3 are core PCP components (Boutin et al., 2012; Goffinet and Tissir, 2017). Celsr3 is expressed in postmitotic neurons and regulates axon guidance in forebrain tracts. Mice with a null mutation in Celsr3 have defects in the anterior commissure and internal capsule (Tissir et al., 2005). Conditional deletions of Celsr3 in the pallium or ganglionic eminences show the requirement of Celsr3 in pyramidal neurons to form the corticospinal tract and in guidepost cells (“corridor cells”) of the ventral telencephalon for the internal capsule formation (Zhou et al., 2008; Zhou et al., 2010). These results suggests that the role of Celsr3 is cell autonomous for corticospinal tract formation, and non-cell autonomous for corticothalamic and thalamocortical projections (Feng et al., 2016). Fzd3, a non-canonical Wnt receptor, is also implicated in the formation of forebrain connections (Wang et al., 2002; Hua et al., 2014). Fzd3 null mice have a similar axonal phenotype as Celsr3. In the telencephalic commissures, the posterior part of the anterior commissure and corpus callosum are missing (Qu et al., 2014). Conditional deletion of Fzd3 in the ventral telencephalon produced aberrant thalamocortical, corticothalamic and corticospinal tracts. These results suggest Fzd3 and Celsr3 share similar mechanism for axon guidance. The role of Vangl2 in axon guidance is more controversial. Studies of the *looptail* (*Lp*) mutants suggest that Vangl2 regulates the formation of commissural axons in the spinal cord (Shafer et al., 2011), brainstem (Fenstermaker et al., 2010), and visual system (Leung et al., 2016). However, Vangl2 knockout mice do not show any axonal defect in the forebrain (Chai et al., 2014; Qu et al., 2014; Chai et al., 2015).

## Cortical Synaptogenesis

Once neurons reach the target area they form synapses. Polarization is essential for asymmetric distribution of proteins between the presynaptic (axon) and postsynaptic compartment (dendrites). Pyramidal neurons receive information mainly from dendrites, which develop small protrusions called dendritic spines, and express receptors that capture and transduce the biochemical signals/neurotransmitters released by the presynaptic compartment.

**FIGURE 3:**
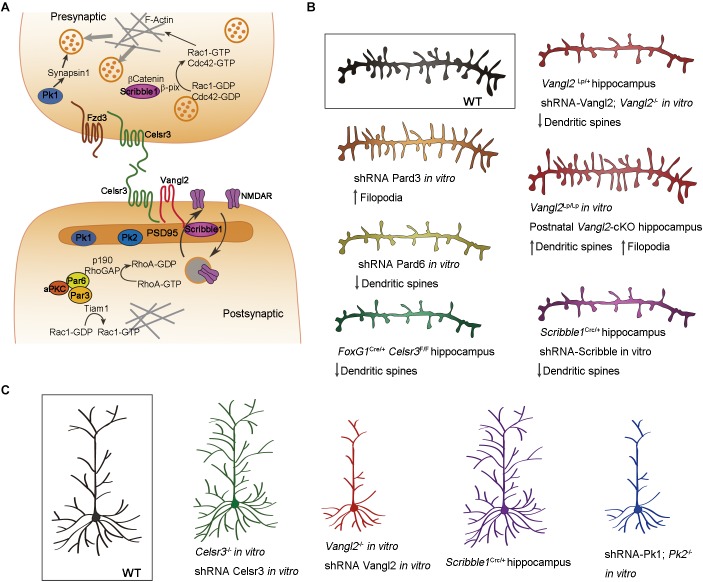
Cell polarity in dendritic morphogenesis. **(A)** Schematic illustration showing the localization of cell polarity proteins in the pre- and postsynaptic compartments of the neuron. Celsr3, Fzd3, and Vangl2 are transmembrane proteins distributed asymmetrically in glutamatergic synapses. Scribble1, Prickle (Pk) and Par complex proteins are intracellular effectors regulating cytoskeleton dynamics important for dendritic spine morphogenesis, neurotransmitter release and synaptic plasticity. **(B)** Implication of cell polarity proteins in dendritic spine morphogenesis. Downregulation of Celsr3, Scribble1, and Par6 generates neurons with less dendritic spines. Par3 downregulation produces more filopodia-like dendritic spines. The effect of Vangl2 depends on the type of mutation and temporal deletion of the gene. **(C)** Schematic illustration showing the implication of cell polarity proteins in dendritic morphogenesis. Downregulation of Celsr3 or Scribble1 causes dendrite overgrowth, whereas downregulation of Vangl2 or Prickle generates less and shorter dendrites. Color code: Celsr3 (green), Scribble1 (purple), Vangl2 (red), prickle (blue), Par3 (orange), Par6 (yellow).

The role of Par proteins in neuronal polarization is well known, but their function in synaptogenesis was for long time neglected. Using interference RNA to downregulate the expression of Par proteins in hippocampal neurons, Zhang and Macara uncovered the important role of Par3 and Par6 in dendritic spine morphogenesis (Zhang and Macara, 2006, 2008). In hippocampal neurons, Par3 is localized in the dendritic spines and regulates the spatial localization and activity of Tiam1. When Par3 is downregulated in neurons, Tiam1 activates Rac1 outside the dendritic spine puncta, producing an increase in the number of immature spines along with a decrease in the number of mature synapses (Zhang and Macara, 2006; Duman et al., 2013) (Figure 3A,B). Par6 is also expressed in dendritic spines and promotes their formation in hippocampal neurons (Zhang and Macara, 2008). As mentioned above, phosphorylation of Par6 at the prospective axon during neuronal polarization activates Smurf1, degrading RhoA and promoting neurite elongation (Ozdamar et al., 2005; Wang H. R. et al., 2006; Cheng et al., 2011). In dendritic spines, Par6 in association with aPKC, inhibits RhoA activity by activating p190 RhoGAP (Figure 3A). Downregulation of Par6 decreases dendritic spine density while its overexpression increases the number of dendritic spines (Zhang and Macara, 2008) (Figure 3B).

The transmembrane PCP proteins are asymmetrically distributed in the pre and postsynaptic compartments. Fzd3 is expressed in the presynaptic bouton, Vangl2 in the dendritic spines, and Celsr3 is expressed in both compartments (Thakar et al., 2017) (Figure 3A). Vangl2 associates with the postsynaptic protein PSD95 and it is implicated in this compartmentalization promoting the endocytosis of N-Cadherin (Yoshioka et al., 2013; Nagaoka et al., 2014, 2015; Nagaoka and Kishi, 2016). Knocking-down Vangl2 in hippocampal neurons impairs the development of dendritic spines and synapses (Hagiwara et al., 2014; Nagaoka et al., 2014; Okerlund et al., 2016). Here again, the results obtained from different alleles and or by different groups are not always consistent. *Vangl2*^-/-^, unlike *Vanl2*^Lp/Lp^, neurons develop less dendritic spines *in vitro*. Likewise, *Vangl2*^Lp/+^ neurons form less spines *in vivo* (Nagaoka et al., 2014; Okerlund et al., 2016), whereas other authors report opposite results for *Vangl2* conditional knockouts (Thakar et al., 2017) (Figure 3B). These discrepancies could be due to differences in the temporal inactivation of Vangl2, dominant negative activity of the *Lp* mutation, or to differences in the role of Vangl2 in dendritic spine formation and pruning. Conditional deletion of *Celsr3* in the embryonic forebrain (*Celsr3*^fl/fl^; *Foxg1-*Cre) produces pyramidal neurons with less dendritic spines in the hippocampus (Feng et al., 2012) (Figure 3B). Celsr3-deficient hippocampal neurons show a reduced number of glutamatergic synapses puncta *in vitro*, and decreased excitatory postsynaptic currents (mEPSC), indicating a reduction in glutamatergic synapses (Thakar et al., 2017). Celsr3 and Vangl2 have also opposing roles in dendritic morphogenesis. Celsr3 restricts dendrite growth through homophilic interactions (Shima et al., 2007; Feng et al., 2012), whereas Vangl2 promotes extension and branching of dendrites (Hagiwara et al., 2014) (Figure 3C). The intracellular effector Scrib1, which is involved in both apicobasal and planar polarity through interaction with Vangl2 and Par proteins (Bilder and Perrimon, 2000; Montcouquiol et al., 2003; Humbert et al., 2006; Kallay et al., 2006; Assemat et al., 2008) localizes at the presynaptic and postsynaptic compartment (Audebert et al., 2004; Sun et al., 2009; Moreau et al., 2010; Richier et al., 2010; Piguel et al., 2014) (Figure 3A). In the presynaptic bouton, Scrib1 is important for clustering synaptic vesicles (Sun et al., 2009). β-catenin is implicated in the localization of Scrib1 in the presynaptic compartment. The complex formed by Scrib1/βCatenin recruits a GEF called β-pix that, in turn, activates Rac1/Cdc42 increasing the polymerization of actin filaments and “sequestering” synaptic vesicles at the presynaptic compartment (Sun and Bamji, 2011) (Figure 3A). Scrib1 has also a role in the formation of dendrites. Hippocampal neurons from Scrib1Crc/+ heterozygous mice (carrying the circletail spontaneous mutation), develop more complex dendritic trees but fewer dendritic spines *in vivo* (Figure 3B,C). The morphology of the postsynaptic protein PSD95 is also altered, with larger PSD domains in Scrib1Crc/+ hippocampal neurons compared with controls (Moreau et al., 2010). The same results arise when Scrib1 is downregulated *in vitro* using shRNA (Moreau et al., 2010) (Figure 3B). Scrib1 is also implicated in the internalization and recycling of NMDA receptors at the postsynaptic plasma membrane (Piguel et al., 2014) (Figure 3A). Scrib1Crc/+ mice exhibit deficits in social interactions but improved learning and memory (Moreau et al., 2010), while postnatal deletion of Scrib1 (Scrib1fl/fl; CamKII-Cre) impairs NMDAR-dependent synaptic plasticity in the hippocampus (Hilal et al., 2017). Overexpression of Pk1 and Pk2, two Vangl-interacting proteins promote neurite outgrowth, and its downregulation produces shorter neurites in neuroblastoma cells (Okuda et al., 2007; Fujimura et al., 2009). Hippocampal neurons from Pk2-/- mice, or transfected with Pk1-shRNA in culture exhibit reduced number and length of dendrites (Liu et al., 2013; Sowers et al., 2013). Pk1 associates with Synapsin1 in the presynaptic compartment and regulates the neurotransmitter release. The mutation of Pk1 impairs synaptogenesis, and Pk1+/- mice exhibit features reminiscent of ASD (Paemka et al., 2013). Pk2 is expressed in the postsynaptic compartment of cortical neurons and promote synapse formation (Okuda et al., 2007; Hida et al., 2011; Sowers et al., 2013). Disrupting Pk2 in mice impairs synaptic transmission and results in “autistic-like” behavior (Sowers et al., 2013).

## Concluding Remarks and Perspectives

A striking feature of cell polarity is that it is intimately linked to adhesion, and an essential issue that requires further scrutiny is how the two processes are orchestrated in time and space. Indeed, like polarity, adhesion is essential to progenitor cell proliferation, neuronal differentiation and migration, axon and dendrite specification, and synapse formation. Adhesion proteins sit at the center of AJCs. These structures serve as molecular platforms that assemble polarity protein complexes during cell division and orient the mitotic spindle, thereby affecting fate determination of neural progenitors and differentiation of neural cells. In the case of PCP, the Celsr (Cadherin, EGF like, Laminin G like, seven-pass G-type receptor) proteins, which are natural chimeras of adhesion molecules and G protein-coupled receptors (Tissir and Goffinet, 2010; Boutin et al., 2012; Tissir and Goffinet, 2013a), serve both adhesive and polarity functions, and are therefore ideal candidate to coordinate the two processes. Celsr1 is specifically expressed in aRG and its loss-of-function compromises their fate decision. In absence of Celsr1 aRG undergo more proliferative divisions, expanding the pool of progenitors at the expense of neurons (Boucherie et al., 2018). In the case of apical–basal polarity N-cadherin appears as a central protein. The delamination of progenitors and transition from bipolar to multipolar shape require disassembly of AJCs and involve recycling of cadherins (N-cadherin is downregulated, Celsr1 is silenced), and maybe also atypical cadherins Fat and Dchs, and this correlates with centrosome relocation and cytoskeleton rearrangement. Cortical neurons recover a bipolar shape as they move out of IZ, and this implies cooperation of polarity and adhesion proteins to reorganize the cytoskeleton and form new adhesions between migrating neurons and aRG. The same mechanisms are used repetitively to assemble/disassemble cell–cell adhesions as locomotion proceeds along aRG fibers. Adhesion proteins N-cadherin, Astrotactin 1 and 2, integrins, Connexins 26 and 43, Jam3, as well as focal adhesion molecules FAK and paxillin have been involved. Axon-dendrite specification is concomitant with glial-guided locomotion, and the underlying mechanisms are in large extent thought to be indistinguishable of those that regulate bipolarity. Axon-dendrite specification correlates with the upregulation of Celsr3 and involves both Celsr2 and Celsr3.

The crosstalk between polarity and adhesion is not restricted to polarization of neural progenitors and migration of immature neurons during embryogenesis. Polarity and adhesion proteins are “recycled” in maturating neurons where they regulate synaptogenesis and connectivity. Their interaction is key to target selection and formation of presynaptic versus postsynaptic specifications. The role core PCP components in synapse formation and function/dysfunction start to emerge in the last few years. Mutations and genetic variants in PCP genes were identified in patients with neurological disorders. Fzd3 has been associated with schizophrenia. Different Fzd3 point mutations were found in Chinese and Japanese populations suffering from schizophrenia (Katsu et al., 2003; Yang et al., 2003; Zhang et al., 2004; Kang et al., 2011), even though other studies reported weak or no association (Ide et al., 2004; Wei and Hemmings, 2004; Hashimoto et al., 2005; Jeong et al., 2006; Reif et al., 2007; Pantavou et al., 2016). Analysis of *de novo* gene disruptions in patients with the Tourette syndrome (TS), pointed to Celsr3 as a high-confidence risk gene (Willsey et al., 2017; Wang et al., 2018). Interestingly, the same study found mutations in other polarity genes suggesting that dysfunction in polarity contribute to TS (Wang et al., 2018). Prickle proteins are involved in dendritic morphology and synaptogenesis and Pk mutant mice exhibit ASD-like behavior. PK1 mutations have been reported in patients suffering from myoclonic epilepsy, ataxia, ASD, polymicrogyria, agenesis of the corpus callosum and global developmental delay (Bassuk et al., 2008; Tao et al., 2011; Bassuk and Sherr, 2015; Todd and Bassuk, 2018). PK2 mutation is associated with ASD, schizophrenia, bipolar disorder and depression (Okumura et al., 2014; Chen et al., 2017), but the link between PK2 and epilepsy remains unclear (Mahajan and Bassuk, 2016; Sandford et al., 2016). SCRIBBLE, another PCP protein involved in synaptogenesis, is an ASD candidate (Neale et al., 2012; O’Roak et al., 2012; Battaglia et al., 2013). Contrary to PCP, no link between apical–basal polarity genes and neurological/psychiatric disorders has been documented thus far. However, searching the DECIPHER database we found some hints of this link. A patient with a single nucleotide variation (SNV) in PARD6 gamma in heterozygosis presents intellectual disability (Decipher ID: 351821), and other patients with duplications and deletions in the same gene have similar phenotypes (Decipher ID: 259718, 288221, and 290431). A small deletion in PARD3 gene was identified in patients with intellectual disability, delayed speech, and autism (Decipher ID: 256704 and 366388). A patient suffering intellectual disability carry a specific deletion in the aPKC beta (Decipher ID: 290292).

Conditional gene manipulation, advanced downregulation techniques, and genome editing in animal models, along with high throughput genome sequencing will eventually help assess late functions of apical–basal polarity genes and link their dysfunction to neurological and psychiatric disorders.

## Author Contributions

All authors listed have made a substantial, direct and intellectual contribution to the work, and approved it for publication.

## Conflict of Interest Statement

The authors declare that the research was conducted in the absence of any commercial or financial relationships that could be construed as a potential conflict of interest.
